# Supplementary material for: Polymeric Encapsulate of Streptomyces Mycelium Resistant to Dehydration with Air Flow at Room Temperature
Source: Polymers (Basel). 2022 Dec 31;15(1):207. doi: 10.3390/polym15010207 (PMC9823993; doi:10.3390/polym15010207)
Supplement: Supplementary file 1 [file polymers-15-00207-s001.zip › polymers-2065389-supplementary.pdf]

Supplementary Tables and Figures

Table S1. Formulations of *Streptomyces* strains

Strain	Encapsulated biomass	Formulation	Supplements	CFU mL^{-1} (or g^{-1}) before/after encapsulation	shelf-life	Storage at	Ref.
Streptomyces sp. T-741	spores	Lyophilization and freezing in liquid nitrogen	sucrose (10 %), skimmed milk (5.5 %) and gelatin (1.5 %)	80-90%	8 years	4°C	[21]
Streptomyces rochei	spores	wettable talcum powder, sodium alginate pellets and sodium alginate-clay pellets *. Calcium alginate capsules were dried	1.5 g of calcium carbonate and 10 g of carboxymethyl cellulose	2.1×10^5 CFU g^{-1} to 1.9×10^5 CFU g^{-1} /2×10^6 CFU g^{-1} to 1.7×10^6 CFU g^{-1}; $2x10^6$ CFU ml^{-1} to 3.7×10^5 CFU g^{-1} (after drying 92%; 91%; 89%)			[27]
Streptomyces cameroonensis	sporal solution	Talcum powder and cassava starch (a 0.8 mm sieve)	15 g calcium carbonate and 10 g methyl cellulose	10^9 CFU/ml (400 mL); 1.07×10^6 CFU g^{-1}	6 months	4°C	[16]
Streptomyces sp. Di-944	lyophilized fragments of viable vegetative filaments	Alginate beads, durum flour (starch) granules, and talcum powder formulation		(6.9×10^4 and 7.3×10^2 viable propagules/g) 2×10^7CFU g^{-1}; 1.2×10^5 CFU g^{-1}; 7×10^3 CFU g^{-1}	(12-24 weeks) 14-10 weeks (good)	4°C	[44]
Streptomyces griseus	vegetative cells	sodium alginate/ kaolin clay/ alginate–kaolin combination		10^6 CFU g^{-1} soil/ $10^{6.12}$ / $10^{6.16}$ CFU g^{-1}			[13]
Actinobacterias	vegetative cells	cryopreservation and dry-freezing; clay, silica and sand	cryoprotectants, glycerol at 20% and 30% (v/v) to freeze and 18% skim milk (w/v) for dry-freezing	10^8 cells mL^{-1} (glycerol at 30% was the best)	1 year	4°C	[18]
Streptomyces fradiae NKZ-259	Vegetative cells	wettable starch-kaolin powder		(5.4×10^6 CFU/mL) 73.74 %	4 months	4°C	[15]

*Texts were marked in color to relate data by line.

Table S2. Encapsulated and dehydrated formulations of bacteria with application in soil fertilization.

Drying method	Formulation	Drying time (h)	Microorganisms used	Wet beads diameter	CFU g^{-1} Initial(wet)/final (dry)	Ref.
Dry air at 38°C	Alginate beads	48 h/(10g beads)	Azospirillum brasilense Cd (ATCC 29710	100 - 200 µm	$2x10^{11/}3x10^{9}$	[22]
Laminar flow hood at 25°C	Alginate beads	20 h)/50g beads	Cells of Pseudomonas fluorescens	1mm	$4.5x10^{11}/4.7x10^{8}$	[23]
Dried in an oven at 35°C	Alginate and starch	24 h	*Azospirillum brasilense* Sp245	2.3 mm	$2.8x10^{10}/6.5x10^{6}$	[24]
Drying chamber (desiccator) 23°C	Alginate and protective ingredients	72 h,	*Lactobacillus rhamnosus* R0011	2.5 mm	$1x10^{8}$/2.5% or 56% with maltodextin and y glycerol	[25]
Spray-dried inlet air temperature 130°C; outlet temperature 49°C.	Algnate powder		Methylobacterium radiotolerans	powder	$1x10^{11}/1x10^{10}$	[26]
Laminar flow hood	Sodium alginate pellets	Overnight	Spores of the strain PTL2 (S. rochei NBRC 12908T)	3mm	$2x10^{6}/4.5×10^{5}$	[27]
Dried at room temperature	Microcapsuled	to obtain constant weight	*Klebsiella oxytoca* Rs-5	1.10 mm	$10^{14}/10^{10}$	[28]
Oven maintained at 40°C	Alginate-starch-bentonite	48 h	Raoultella planticola Rs-2	2.2- 3.6mm	$9.11x10^{12}/5.9x10^{6}$	[29]
Circulating air oven at 40°C	Starch–alginate beads (10:1.5 %w/v)	12 hours	Aspergillus flavus spores	1.95mm	7.5 log/4.5 log	[30]

* References included at the end of this document.

Table S3. Characteristics of drying methods most frequently applied during formulation development of bacteria inoculants [14].

	Freeze drying	Spray drying	Fluidized bed-drying	Vacuum-drying	Air-drying
Minimum sample size	μL - mL	< 100 mL	< 100 mL	μL - mL	μL - mL
Typical temperature range	< 0°C	inlet 100-200°C; outlet 60-85°C	30-35°C	40-70°C	25-35°C
Matrix compatibility	No limitations	Liquid matrix	Liquid matrix sprayed onto carriers; pre-dried granular matrix	No limitations	No limitations
Out form	Cake – further processing required	Small sized particles	Medium sized, particles, granules	Cake – further processing required	Cake – further processing required
Typical drying time	Hours-days	Seconds-minutes	Minutes-hours	Hours-days	Hours-days
Operation	Straight forward	Challenging	Challenging	Straight forward	Challenging

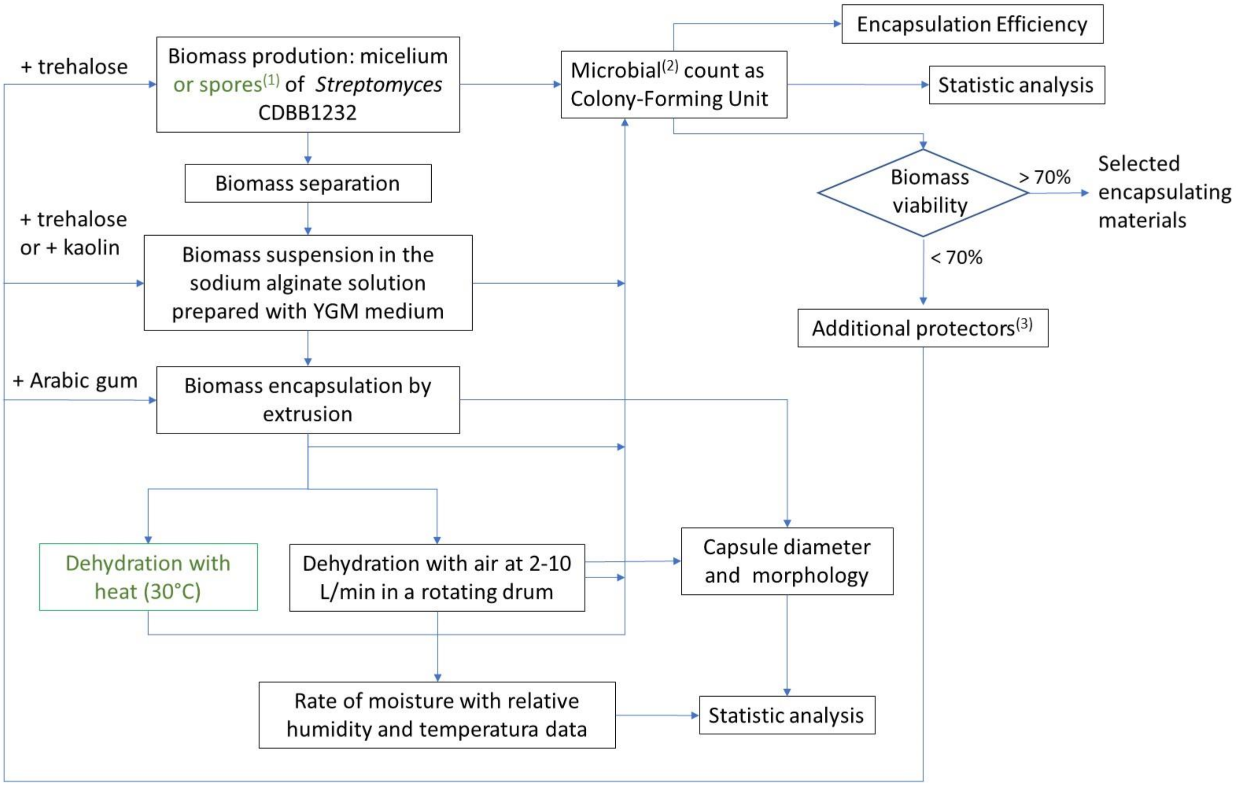

(1) Control experiments are identified in green.
(2) The spore count was determined in a Neubauer chamber.
(3) Biological material released from the alginate matrix and suspended in sterilized distilled water or 1% sodium citrate solution.
(4) Studies carried out with data reported in the literature

Figure S1. Experimental methodology
